# Occupational exposure to chlorinated solvents and risk of head and neck cancer in men: a population-based case-control study in France

**DOI:** 10.1186/s12940-017-0286-5

**Published:** 2017-07-24

**Authors:** Christine Barul, Aurore Fayossé, Matthieu Carton, Corinne Pilorget, Anne-Sophie Woronoff, Isabelle Stücker, Danièle Luce, ICARE study group, Anne-Valérie Guizard, Arlette Danzon, Anne-Sophie Woronoff, Michel Velten, Antoine Buemi, Émilie Marrer, Brigitte Trétarre, Marc Colonna, Patricia Delafosse, Paolo Bercelli, Florence Molinié, Simona Bara, Bénédicte Lapotre-Ledoux, Nicole Raverdy, Sylvie Cénée, Oumar Gaye, Florence Guida, Farida Lamkarkach, Loredana Radoï, Marie Sanchez, Isabelle Stücker, Matthieu Carton, Diane Cyr, Annie Schmaus, Joëlle Févotte, Corinne Pilorget, Gwenn Menvielle, Danièle Luce

**Affiliations:** 1 INSERM U 1085, Institut de Recherche en Santé, Environnement et Travail (IRSET), Pointe-à-Pitre, France; 2 Paris Saclay University, Villejuif, France; 3 INSERM, Population-based Epidemiologic Cohorts Unit, UMS 011 Villejuif, France; 4 The French Public Health Agency, Saint Maurice, France; 5 Univ Lyon, Claude Bernard Lyon1 University, Ifsttar, UMRESTTE, UMR T_9405, F-69373 Lyon, France; 6 Registre des tumeurs du Doubs et du Territoire de Belfort CHRU, Besançon, France; 7 INSERM, CESP Centre for Research in Epidemiology and Population Health, Environmental Epidemiology of Cancer Team, Villejuif, France; 8 Address: INSERM U1085-IRSET, Faculté de Médecine, Campus de Fouillole, BP-145, 97154 Pointe-à-Pitre, France

**Keywords:** Cancer, Occupation, Solvents, Epidemiology

## Abstract

**Background:**

Few epidemiological studies have investigated the link between occupational exposure to solvents and head and neck cancer risk, and available findings are sparse and inconsistent. The objective of this study was to examine the association between occupational exposure to chlorinated solvents and head and neck cancer risk.

**Methods:**

We analyzed data from 4637 men (1857 cases and 2780 controls) included in a population-based case-control study, ICARE (France). Occupational exposure to five chlorinated solvents (perchloroethylene [PCE], trichloroethylene [TCE], methylene chloride [MC], chloroform [CF], and carbon tetrachloride [CT]) was assessed through job-exposure matrices. Odds ratios (ORs) and confidence intervals (95% CI) were estimated by unconditional logistic regression, adjusted for age, tobacco smoking, alcohol consumption, asbestos exposure, and other potential confounders.

**Results:**

We observed no association between chlorinated solvent exposure and head and neck cancer risk, despite a non-significant increase in risk among subjects who had the highest cumulative level of exposure to PCE, (OR = 1.81; 95% CI = 0.68 to 4.82). In subsite analysis, the risk of laryngeal cancer increased with cumulative exposure to PCE (p for trend = 0.04). The OR was 3.86 (95% CI = 1.30 to 11.48) for those exposed to the highest levels of PCE. A non-significant elevated risk of hypopharyngeal cancer was also observed in subjects exposed to the highest levels of MC (OR = 2.36; 95% CI = 0.98 to 5.85).

**Conclusion:**

Our findings provide evidence that high exposure to PCE increases the risk of laryngeal cancer, and suggest an association between exposure to MC and hypopharyngeal cancer. Exposure to other chlorinated solvents was not associated with the risk of head and neck cancer.

**Electronic supplementary material:**

The online version of this article (doi:10.1186/s12940-017-0286-5) contains supplementary material, which is available to authorized users.

## Background

Until recently, chlorinated solvents have been widely used in practically all branches of modern industry. Their use has since decreased in response to various factors, including increasing knowledge pertaining to their toxicity and environmental impact [1–4]. Several chlorinated solvents are known or suspected carcinogens. Commonly used for metal cleaning and degreasing, trichloroethylene (TCE) was classified in 2012 as carcinogenic for humans (group 1) by the International Agency for Research on Cancer (IARC), based on sufficient evidence that it causes kidney cancer and limited evidence for liver cancer and non-Hodgkin lymphoma (NHL) [5]. The same year, perchloroethylene (PCE), widely used in the dry cleaning sector, was classified as a probable carcinogen (group 2A), based on limited evidence of an increased risk of bladder cancer [5]. More recently, methylene chloride (MC) was also classified as a probable carcinogen (group 2A) based on limited evidence that it causes biliary tract cancer and NHL [6]. Moreover, chloroform (CF) and carbon tetrachloride (CT) are considered to be possibly carcinogenic (group 2B) [7].

Few epidemiological studies have examined the link between occupational exposure to chlorinated solvents and head and neck cancer risk, and available findings are sparse and inconsistent. Cohort studies could not control for smoking and alcohol use and lacked statistical power to detect moderate increases in risk, because they were often conducted in countries where the incidence of head and neck cancer is low. However, non-significant increases in the risk of oral, pharyngeal, and laryngeal cancer were found in several cohorts of workers exposed to TCE or PCE [8, 9]. Case-control studies included proper adjustment for confounders, but exposure to solvents was often not well characterized. Exposure to solvents in general has been found to be associated with oral and pharyngeal [10] or laryngeal and hypopharyngeal [11] cancer risk, although others did not find an association [12]. Shangina et al. found a significantly increased risk of laryngeal cancer associated with chlorinated solvent exposure [13] and Vaughan et al. found a non-significant increased risk of oral, pharyngeal, and laryngeal cancer among subjects exposed to PCE [14].

No study has yet examined the association between exposure to several specific chlorinated solvents and head and neck cancer risk, by cancer site. In a previous analysis of our study population by occupation, we observed increased risks of head and neck cancer in dry cleaners and metal workers, suggesting a possible role of exposure to chlorinated solvents [9]. Here, our objective was to investigate the association between exposure to five chlorinated solvents and head and neck cancer risk, using data from a large population-based case-control study, the ICARE study.

## Methods

### Study design and population

The ICARE study is a French multicenter, population-based case-control study, conducted between 2001 and 2007 in ten geographical areas covered by a cancer registry. The study design has been described in detail elsewhere [15]. Incident cases of head and neck cancers were identified in almost all healthcare establishments in each area, in collaboration with the cancer registries. All new patients with histologically confirmed primitive tumors of the oral cavity, pharynx, sinonasal cavities, and larynx (International Classification of Diseases for Oncology 3rd revision (ICD-O-3) codes C00-C14; C30-C32), between 18 and 75 years old, who were diagnosed during the study period, were eligible for the study. Selection of the control population was made by incidence density sampling. Controls were frequency-matched to cases by gender and age (<40, 40–54, 55–64, ≥ 65 years old) and further stratification was performed to match the socioeconomic distribution of controls and that of the general population. Among the 4047 eligible head and neck cancer cases, 596 (14.7%) patients could not be located, 299 (7.4%) died before the interview, and 225 (5.6%) could not be interviewed due to poor health. Among the 2927 potential subjects who were contacted, 2415 (82.5%) agreed to participate and were interviewed, on average, within three months of diagnosis [15, 16]. The present study was restricted to men, as women were analyzed separately [17], and to squamous cell carcinomas of the oral cavity (Codes of the International Classification of Diseases for Oncology, third edition (ICD-O-3): oral cavity (C00.3–9; C02.0–3; C03.0–1; C03.9, C04.0–1; C04.8–9; C05.0; C06.0–2; C06.8–9),oropharynx (C01.9; C02.4; C05.1–2; C09.0–1; C09.8–9; C10.0–4; C10.8–9), hypopharynx (C12.9; C13.0–2; C13.8; C13.9), oral cavity or pharynx not otherwise specified or overlapping (C02.8, C02.9, C05.8, C05.9, C14.0, C14.2, C14.8) and larynx (C32.0–3; C 32.8–9). Overall, 1857 male cases (350 with oral cavity cancers, 543 with oropharyngeal cancers, 383 with hypopharyngeal cancers, 454 with laryngeal cancers, 127 with cancer of the oral cavity or pharynx not otherwise specified or overlapping) and 2780 male controls were included.

### Data collection

Trained interviewers conducted face to face interviews using a standardized questionnaire. Data included sociodemographic characteristics, smoking and alcohol consumption history, and a detailed lifetime occupational history, which covered all jobs held for at least one month. For subjects who had difficulty answering because of sickness or tiredness, a shorter version of the questionnaire was used for either in-person interview or to interview a next-of-kin. This shorter version included mainly information on smoking, alcohol consumption, and occupational history and was used for 11% of the cases and 2% of the controls. Occupations and branches of industry were coded by trained coders, blinded to the case-control status of the subjects, according to the International Standard Classification of Occupations (ISCO) [18] and the French Nomenclature of Activities (NAF) [19].

### Assessment of occupational exposure

Occupational exposure to TCE, PCE, MC, CF and CT was assessed using job-exposure matrices (JEMs), developed in the context of the Matgéné program [1]. For each combination of ISCO and NAF codes, the JEMs provided three indices of exposure: (i) the probability of exposure expressed as the percentage of exposed workers (categorized into not exposed, 1–10,11–20,21–30, up to 91–100%), (ii) the intensity of exposure (for PCE, TCE and MC: not exposed, 5–25, 26–50, 51–100, >100 ppm; for CT and CF: not exposed, very low, low, medium, and high) and (iii) the frequency of exposure (not exposed, 1–10, 11–20, 21–30, up to 91–100% of working time). In addition, a specific JEM assessed the probability of exposure to at least one of these five chlorinated solvents, together with the average level of exposure during a usual working day. Exposure indices were provided for different calendar periods (1950–1969, 1970–1984, 1985–1994, >1995) to account for changes in working practices and regulation over time. As an example, in France, for methylene chloride, exposure limits decreased from 100 ppm in 1985 to 50 ppm in 1995, and a general improvement in working conditions was assumed to occur at the beginning of the 1970’s. After linking these indices with lifetime occupational history, the following exposure variables were obtained for each subject: ‘ever/never’ exposed to a specific chlorinated solvent (‘ever’ defined as having worked in at least one job with a probability of exposure greater than zero), total duration of exposure, and cumulative exposure index (CEI). CEIs were calculated by summing the product of the exposure probability, frequency, intensity, and duration of each job period, over the entire work history, using the central value of the classes. For exposure to at least one solvent, as the JEM provides the average level and the probability of exposure, the CEI was calculated by summing the product of exposure probability, average level and duration. We then categorized these variables. The duration of exposure to solvents was categorized into four classes: ‘never exposed’, and three categories according to approximate tertiles of the distribution among exposed controls, ‘short’, ‘intermediate’, and ‘long’ exposure. To examine potential effects of the highest exposures to chlorinated solvents, CEIs were categorized as follows: ‘never exposed’, and three categories according to the percentiles of the distribution among exposed controls (low: < 50th; medium: 50th–90th; high: > 90th). The CEI categories “medium” and “high” for exposure to CF and CT were combined because of the small number of subjects in these groups.

We estimated the prevalence of lifelong exposure to chlorinated solvents by weighting the number of subjects exposed in each class of maximum probability by the central value of the class. Non-exposed subjects were then recalculated accordingly. Occupational exposure to asbestos was assessed through a specific JEM [20].

### Other variables

Covariates included age at interview in categories [years] (< 40; [40–49]; [50–59]; [60–69]; ≥ 70), area of residence, alcohol consumption in categories [glasses/day] (< 0.03: occasional consumption; [0.03–2.00]; [2.01–5.00]; [5.01–8.00]; [8.01–12.00]; > 12), daily amount of tobacco in categories [g/day] (0; [0–10] [11–20]; [21–25]; ≥ 25), duration of tobacco smoking in categories [years] (0; [0–20]; [21–30]; [31–40]; ≥ 40), smoking status (‘never’, ‘former’ [time since stopping smoking >2 years before the interview], and ‘current’ [time since stopping smoking ≤2 years before the interview]). We also fitted models with and without cumulative asbestos exposure in four categories (never exposed, and tertiles according to the distribution among exposed controls).As the inclusion of asbestos exposure resulted in changes in ORs for most solvents and no change in OR point estimate without loss of precision for the others, we present below the models adjusted for asbestos exposure (to assess the magnitude of confounding, OR estimates without adjustment for asbestos are presented in Additional file 1: Table S1).

### Statistical analysis

Multivariable unconditional logistic regression models were used to model associations between chlorinated solvents and head and neck cancer risk. Odds-ratios (ORs) and corresponding 95% confidence intervals (95% CI) were adjusted for previously described covariates. Each solvent was analyzed separately. We also estimated mutually adjusted ORs in a model including all solvents.

Tests for linear trends were performed by modelling the median of each category as a continuous variable.

Additional analyses were performed in separate models adjusting for educational level or occupational class of the longest job held. We also conducted analyses by cancer site (oral cavity, oropharynx, hypopharynx, oral cavity or pharynx not specified, and larynx) and subsites using polytomous logistic regression. Finally, to determine whether joint-exposure to several chlorinated solvents was associated with head and neck cancer risk, we examined the risk associated with combinations of chlorinated solvents with sufficient numbers (at least 10 exposed cases), using “never exposed to any chlorinated solvents” as the reference category.

For each solvent, we also assessed potential interactions with smoking, alcohol drinking and asbestos exposure by including cross-product terms in the models. None of the interactions were statistically significant, and the results were not presented. We also conducted sensitivity analyses excluding subjects with the shorter version of the questionnaire, and the results were similar (data not shown).

## Results

The age distribution differed slightly between cases and controls, but the mean age was similar. Cases had a lower level of education than controls and were more often blue collar workers. As expected, cases were more often smokers than controls and had a higher level of alcohol consumption (Table 1).

**Table 1:** Main characteristics of cases and controls

	Cases (*n* = 1857)	Controls (*n* = 2780)
	n (%)	n (%)
Age (years)		
< 40	24 (1.3)	76 (2.7)
[40;49]	286 (15.4)	555 (20.0)
[50;59]	786 (42.3)	825 (29.7)
[60;69]	548 (29.5)	939 (33.8)
≥ 70	213 (11.5)	385 (13.9)
Missing	0 (0.0)	0 (0.0)
Mean ± sd	58.01 ± 8.50	58.07 ± 9.92
Level of education		
Primary	544 (29.3)	521 (18.7)
Vocational secondary	761 (41.0)	1081 (38.9)
Secondary	120 (6.5)	310 (11.2)
University	163 (8.8)	752 (27.1)
Other	26 (1.4)	19 (0.7)
Missing	243 (13.1)	97 (3.5)
Socioeconomic status		
Farmers	49 (2.6)	168 (6.0)
Self employed workers	117 (6.3)	152 (5.5)
Managers	115 (6.2)	544 (19.6)
Intermediate occupations	191 (10.3)	564 (20.3)
Employees	190 (10.2)	297 (10.7)
Blue Collar workers	1175 (63.3)	1053 (37.9)
Missing	20 (1.1)	2 (0.1)
Alcohol consumption (glasses per day)		
[0.00; 0.03]	73 (3.9)	206 (7.4)
[0.03; 2.00]	235 (12.7)	1190 (42.8)
[2.00; 5.00]	440 (23.7)	849 (30.5)
[5.00; 8.00]	384 (20.7)	305 (11.0)
[8.00; 12.00]	328 (17.7)	134 (4.8)
> 12.00	331 (17.8)	73 (2.6)
Missing	66 (3.6)	23 (0.8)
Smoking status		
Never	53 (2.9)	753 (27.1)
Former	481 (25.9)	1271 (45.7)
Current	1316 (70.9)	751 (27.0)
Missing	7 (0.4)	5 (0.18)
Smoking frequency (grams/day)		
Never smoker	53 (2.9)	753 (27.1)
[0.00; 10.00]	205 (11.0)	668 (24.1)
[10.00;20.00]	565 (30.4)	725 (26.1)
[20.00; 25.00]	372 (20.0)	261 (9.4)
> 25.00	578 (31.1)	301 (10.8)
Missing	84 (4.5)	72 (2.6)
Smoking duration (years)		
Never smoker	53 (2.8)	753 (27.1)
[0; 20]	115 (6.2)	760 (27.3)
[20; 30]	337 (18.2)	529 (19.0)
[30; 40]	762 (41.0)	439 (15.8)
> 40	575 (31.0)	291 (10.5)
Missing	15 (0.8)	8 (0.3)

Concerning lifetime exposure to chlorinated solvents, the most prevalent exposure was to TCE with 8.76% of cases and 7.53% of controls exposed followed by MC with 1.43% of cases and 1.14% of controls exposed. The prevalence of exposure to PCE, CF, and CT was lower with 0.44%, 0.09%, and 0.06% of cases, and 0.28%, 0.16%, and 0.09% of controls, respectively. Exposures to solvents were correlated, and were also correlated with exposure to asbestos (Additional File 1: Fig. S1).

Individual analysis of the solvents (Table 2) showed no significant association between ever exposure, duration of exposure, or cumulative exposure to PCE, TCE, MC, CF, or CT and head and neck cancer risk. However, we observed a non-significant increased risk among subjects who had the highest cumulative levels of exposure to PCE or MC relative to never exposed subjects (OR = 1.81, 95% CI [0.68 to 4.82] and OR = 1.42, 95% CI [0.70 to 2.87], respectively).

**Table 2:** Association between head and neck cancer and exposure to chlorinated solvents

	PCE			TCE			MC			CF			CT			At least one of these five chlorinated solvents		
Exposure	Co	Ca	OR^a^ [95%CI]	Co	Ca	OR^a^ [95%CI]	Co	Ca	OR^a^ [95%CI]	Co	Ca	OR^a^ [95%CI]	Co	Ca	OR^a^ [95%CI]	Co	Ca	OR^a^ [95%CI]
Never	2581	1635	1	1686	938	1	2432	1508	1	2619	1691	1	2622	1686	1	1645	930	1
Ever	89	70	1.04 [0.69 to 1.59]	989	770	0.93 [0.77 to 1.36]	238	197	0.91 [0.69 to 1.18]	51	14	0.58 [0.27 to 1.26]	48	19	0.60 [0.30 to 1.19]	1030	778	0.91 [0.75 to 1.10]
Duration^b^																		
Short	41	31	1.05 [0.56 to 1.94]	289	215	0.86 [0.66 to 1.14]	93	88	0.96 [0.65 to 1.43]	21	7	0.76 [0.26 to 2.20]	25	10	0.56 [0.22 to 1.43]	291	213	0.87 [0.66 to1.14]
Intermediate	25	23	1.27 [0.60 to 2.74]	390	290	0.97 [0.76 to 1.24]	66	46	0.73 [0.44 to 1.22]	13	2	0.20 [0.03 to 1.34]	10	3	0.36 [0.06 to 2.00]	409	295	0.93 [0.73 to 1.19]
Long	23	16	0.83 [0.37 to 1.88]	310	265	0.95 [0.73 to 1.23]	79	63	0.97 [0.62 to 1.52]	17	5	0.69 [0.18 to 2.60]	13	6	0.87 [0.25 to 2.97]	330	270	0.93 [0.72 to 1.20]
p for trend			0.88			0.85			0.67			0.23			0.38			0.69
CEI																		
Low	44	36	1.15 [0.64 to 2.07]	497	391	0.95 [0.75 to 1.19]	114	107	1.03 [0.71 to 1.48]	25	5	0.56 [0.16 to 1.90]	24	7	0.44 [0.15 to 1.29]	518	397	0.95 [0.75 to 1.19]
Medium	34	20	0.65 [0.31 to 1.36]	397	313	0.95 [0.74 to 1.21]	100	63	0.65 [0.43 to 1.00]	26	9	0.60 [0.22 to 1.61]	24	12	0.74 [0.30 to 1.83]	408	291	0.85 [0.67 to 1.10]
High	11	14	1.81 [0.68 to 4.82]	95	66	0.82 [0.54 to 1.23]	24	27	1.42 [0.70 to 2.87]							104	90	0.94 [0.64 to 1.38]
p for trend			0.48			0.38			0.97			0.05			0.39			0.51

*Abbreviations PCE* perchloroethylene, *TCE* trichloroethylene, *MC* methylene chloride, CF chloroform, *CT* carbon tetrachloride, *OR* odds ratio, *CI* confidence interval, *CEI* cumulative exposure index, *Ca* cases, *Co* controls

^a^OR adjusted for age at interview, residence area, alcohol consumption, smoking status, frequency and duration of smoking, exposure to asbestos

s^b^Categories of duration of exposure to solvents were defined as follows: for PCE: short: < 5, intermediate: 5–15, long: > 15; for TCE and ‘at least one of these five chlorinated solvents’: short: <5, intermediate: 5–20, long: > 20; for MC and CT short: < 5, intermediate: 5 to 15, long: > 15; for CF short: < 10, intermediate: 10 to 20, long: > 20

Further adjustment for the level of education or occupational class did not substantially modify the results (see Additional file 1: Tables S2 and S3).

The results remained globally unchanged when we included all solvents in the model (Table 3).

**Table 3:** Association between head and neck cancer and exposure to chlorinated solvents. Mutually adjusted ORs

	PCE			TCE			MC			CF			CT		
Exposure	Co	Ca	OR^a^ [95%CI]	Co	Ca	OR^a^ [95%CI]	Co	Ca	OR^a^ [95%CI]	Co	Ca	OR^a^ [95%CI]	Co	Ca	OR^a^ [95%CI]
Never	2581	1635	1	1686	938	1	2432	1508	1	2619	1691	1	2622	1686	1
Ever	89	70	1.17 [0.73 to 1.87]	984	767	0.93 [0.76 to 1.14]	238	197	0.95 [0.69 to 1.31]	51	14	0.80 [0.25 to 2.52]	48	19	0.69 [0.25 to 1.90]
CEI															
Low	44	36	1.20 [0.63 to 2.29]	494	389	0.95 [0.75 to 1.20]	114	107	1.10 [0.72 to 1.69]	25	5	0.70 [0.15 to 3.17]	24	7	0.47 [0.14 to 1.55]
Medium	34	20	0.91 [0.40 to 2.08]	395	312	0.95 [0.73 to 1.23]	100	63	0.68 [0.42 to 1.10]	26	9	0.68 [0.14 to 3.21]	24	12	1.05 [0.24 to 4.58]
High	11	14	2.43 [0.83 to 7.08]	95	66	0.76 [0.49 to 1.19]	24	27	1.41 [0.68 to 2.94]						
p for trend			0.25			0.27			0.33			0.03			0.53

*Abbreviations PCE* perchloroethylene, *TCE* trichloroethylene, *MC* methylene chloride, CF chloroform, *CT* carbon tetrachloride, *OR* odds ratio, *CI* confidence interval, *CEI* cumulative exposure index, *Ca* cases, *Co* controls

^a^OR adjusted for age at interview, residence area, alcohol consumption, smoking status, frequency and duration of smoking, exposure to asbestos, exposure to other chlorinated solvents

Analysis of specific head and neck cancer sites **(**Table 4 **)** showed a significant dose-response relationship between cumulative exposure to PCE and laryngeal cancer risk (p for trend = 0.04), and subjects who had the highest cumulative levels of exposure to PCE had a significantly higher risk of laryngeal cancer (OR = 3.86; 95% CI = [1.30 to 11.48]). The OR was lower than 1 in the medium exposure category, but the confidence intervals around each OR were wide. We used restricted cubic splines (4 knots) to verify the linearity assumption, and no significant departure from linearity was found (*p* = 0.61). In addition, we observed an elevated OR (OR = 2.36; 95% CI [0.98 to 5.85]) for hypopharyngeal cancer among subjects with the highest levels of exposure to MC, although the trend was not significant (*p* = 0.22).

**Table 4:** Association between head and neck cancer sites and exposure to chlorinated solvents

Exposure		Oral cavity		Oropharynx		Hypopharynx		Oral cavity/pharynx NOS		Larynx	
		*n* = 328		*n* = 502		*n* = 338		*n* = 114		*n* = 423	
	Co	Ca	OR^a^	Ca	OR^a^	Ca	OR^a^	Ca	OR^a^	Ca	OR^a^
			[95%CI]		[95%CI]		[95%CI]		[95%CI]		[95%CI]
PCE											
Never	2581	318	1	482	1	324	1	111	1	400	1
Ever			0.69 [0.32 to 1.48]		0.98 [0.54 to 1.78]		1.15 [0.58 to 2.27]		0.63 [0.19 to 2.14]		1.29 [0.73 to 2.28]
* CEI*											
Low	44	4	0.55 [0.18 to 1.69]	12	1.39 [0.65 to 2.97]	8	1.47 [0.61 to 3.51]	2	0.87 [0.19 to 3.97]	10	1.29 [0.59 to 2.85]
Medium	34	6	0.97 [0.35 to 2.67]	4	0.44 [0.14 to 1.40]	5	0.79 [0.26 to 2.36]	0	-	5	0.69 [0.24 to 1.99]
High	11	0	-	4	1.81 [0.49 to 6.62]	1	0.72 [0.08 to 6.18]	1	1.30 [0.14 to 11.75]	8	3.86 [1.30 to 11.48]
p for trend			0.40		0.79		0.79		0.74		0.04
TCE											
Never	1686	171	1	281	1	176	1	69	1	241	1
Ever			1.24 [0.91 to 1.70]		0.99 [0.76 to 1.30]		1.00 [0.73 to 1.37]		0.79 [0.49 to 1.27]		0.95 [0.72 to 1.26]
* CEI*											
Low	497	77	1.00 [0.70 to 1.42]	121	0.99 [0.73 to 1.34]	71	0.86 [0.60 to 1.24]	21	0.68 [0.39 to 1.19]	101	0.99 [0.72 to 1.35]
Medium	397	69	1.20 [0.83 to 1.74]	84	0.88 [0.63 to 1.23]	74	1.08 [0.74 to 1.56]	21	0.83 [0.46 to 1.47]	65	0.78 [0.55 to 1.12]
High	95	11	0.87 [0.43 to 1.76]	18	0.81 [0.45 to 1.76]	17	1.05 [0.57 to 1.96]	4	0.63 [0.21 to 1.89]	16	0.69 [0.38 to 1.28]
p for trend			0.90		0.34		0.60		0.47		0.11
MC											
Never	2432	296	1	443	1	295	1	101	1	373	1
Ever			0.80 [0.51 to 1.24]		0.94 [0.65 to 1.35]		1.02 [0.67 to 1.55]		0.87 [0.45 to 1.69]		1.01 [0.69 to 1.47]
* CEI*											
Low	114	13	0.57 [0.30 to 1.08]	33	1.07 [0.67 to 1.72]	19	0.90 [0.51 to 1.59]	7	1.01 [0.43 to 2.36]	35	1.46 [0.92 to 2.32]
Medium	100	15	0.81 [0.43 to 1.51]	21	0.73 [0.41 to 1.27]	14	0.70 [0.37 to 1.35]	5	0.74 [0.28 to 1.99]	8	0.37 [0.17 to 0.80]
High	24	4	1.19 [0.37 to 3.85]	5	0.88 [0.30 to 2.57]	10	2.36 [0.98 to 5.85]	1	0.72 [0.09 to 5.95]	7	1.51 [0.58 to 3.94]
p for trend			0.93		0.51		0.22		0.63		0.69
CF											
Never	2619	326	1	496	1	334	1	113	1	422	1
Ever			0.44 [0.10 to 2.01]		1.05 [0.39 to 2.84]		1.28 [0.39 to 4.13]		0.73 [0.09 to 5.92]		0.18 [0.02 to 1.38]
* CEI*											
Low	25	1	0.45 [0.05 to 3.91]	1	0.42 [0.05 to 3.57]	2	1.45 [0.27 to 7.83]	0	-	1	0.40 [0.05 to 3.43]
Medium - High	26	1	0.35 [0.04 to 3.43]	5	1.16 [0.37 to 3.71]	2	0.80 [0.16 to 4.00]	1	0.95 [0.11 to 8.15]	0	-
p for trend			0.47		0.27		0.26		0.56		0.01
CT											
Never	2622	327	1	495	1	333	1	112	1	419	1
Ever			0.17 [0.02 to 1.31]		0.92 [0.37 to 2.33]		1.05 [0.36 to 3.03]		0.98 [0.21 to 4.54]		0.38 [0.11 to 1.33]
* CEI*											
Low	24	0	-	3	0.74 [0.19 to 2.91]	2	0.65 [0.13 to 3.38]	1	0.83 [0.10 to 7.31]	1	0.25 [0.03 to 2.02]
Medium	24	1	0.30 [0.04 to 2.42]	4	0.92 [0.27 to 3.12]	3	1.08 [0.28 to 4.20]	1	0.97 [0.12 to 8.06]	3	0.64 [0.17 to 2.43]
High											
p for trend			0.18		0.84		0.98		0.96		0.40
At least one chlorinated solvent											
Never	1645	171	1	276	1	174	1	68	1	241	1
Ever			1.17 [0.86 to 1.60]		0.99 [0.76 to 1.29]		1.00 [0.73 to 1.36]		0.79 [0.50 to 1.27]		0.91 [0.69 to 1.20]
* CEI*											
Low	518	77	0.97 [0.69 to 1.38]	117	0.97 [0.71 to 1.31]	73	0.87 [0.61 to 1.25]	22	0.72 [0.41 to 1.26]	108	1.02 [0.75 to 1.39]
Medium	408	64	1.05 [0.72 to 1.52]	89	0.93 [0.67 to 1.29]	64	0.93 [0.64 to 1.36]	21	0.83 [0.47 to 1.47]	53	0.61 [0.42 to 0.89]
High	104	16	1.05 [0.57 to 1.96]	22	0.82 [0.48 to 1.39]	27	1.38 [0.81 to 2.36]	4	0.50 [0.17 to 1.96]	21	0.80 [0.46 to 1.39]
p for trend			0.75		0.48		0.26		0.27		0.07

*Abbreviations PCE* perchloroethylene, *TCE* trichloroethylene, *MC* methylene chloride, CF chloroform, *CT* carbon tetrachloride, *OR* odds ratio, *CI* confidence interval, *CEI* cumulative cxposure index, *Ca* cases, *Co* controls

^a^OR adjusted for age at interview, residence area, alcohol consumption, smoking status, frequency and duration of smoking, exposure to asbestos

The risk of laryngeal cancer associated with the highest level of cumulative exposure to PCE remained significantly elevated when adjusted for occupational class (OR = 3.09; 95% CI [1.05 to 9.13]) or level of education (OR = 3.42; 95% CI [1.09 to 5.77]). However, adjustment for occupational class and level of education for high levels of exposure to MC decreased the OR of hypopharyngeal cancer (OR = 2.08; 95% CI [0.86 to 5.04] and OR = 1.57; 95% CI [0.60 to 4.12], respectively).

We found no significant association between combinations of chlorinated solvents with at least 10 exposed cases and head and neck cancer risk, or specific subsites (Table 5). However, although we observed no increase in laryngeal cancer risk for subjects who were exposed to only TCE (OR = 0.84; 95% CI [0.63 to 1.12]), or the combination of TCE and MC (OR = 0.95; 95% CI [0.59 to 1.53]), the risk of laryngeal cancer was non-significantly elevated for subjects who were exposed to the combination of TCE, PCE, and MC (OR = 1.32; 95% CI [0.68 to 2.55]).

**Table 5:** Association between head and neck cancer and exclusive exposure to combinations of chlorinated solvents

			Head and neck	Oral cavity		Oropharynx		Hypopharynx		Oral cavity /pharynx NOS		Larynx	
			*n* = 1693	*n* = 326		*n* = 497		*n* = 336		*n* = 114		*n* = 420	
	Co	Ca	OR^a^ [95%CI]	Ca	OR^a^ [95%CI]	Ca	OR^a^ [95%CI]	Ca	OR^a^ [95%CI]	Ca	OR^a^ [95%CI]	Ca	OR^a^ [95%CI]
Not exposed to any chlorinated solvent	1645	930	1	171	1	276	1	174	1	68	1	241	1
TCE	761	557	0.93 [0.77 to 1.13]	124	1.18 [0.87 to 1.59]	159	0.93 [0.71 to 1.21]	117	0.99 [0.73 to 1.35]	30	0.70 [0.42 to 1.14]	127	0.84 [0.63 to 1.12]
TCE, PCE	19	12	0.90 [0.36 to 2.23]	1	0.39 [0.05 to 3.19]	4	1.13 [0.33 to 3.50]	2	0.99 [0.20 to 4.94]	2	2.33 [0.46 to 11.67]	3	0.90 [0.24 to 3.16]
TCE, MC	122	131	0.91 [0.65 to 1.27]	21	0.81 [0.47 to 1.40]	39	0 .93 [0.59 to 1.46]	28	0.99 [0.60 to 1.65]	11	1.06 [0.51 to 2.20]	32	0.95 [0.59 to 1.53]
TCE, PCE, MC	62	49	1.05 [0.64 to 1.73]	9	1.00 [0.45 to 2.26]	13	0.91 [0.45 to 1.84]	10	1.11 [0.51 to 2.43]	1	0.26 [0.03 to 2.02]	16	1.32 [0.68 to 2.55]

*Abbreviations PCE* perchloroethylene, *TCE* trichloroethylene, *MC* methylene chloride, CF chloroform, *CT* carbon tetrachloride, *OR* odds ratio, *CI* confidence interval, *CEI* cumulative cxposure index, *Ca* cases, *Co* controls

^a^OR adjusted for age at interview, residence area, alcohol consumption, smoking status, frequency and duration of smoking, exposure to asbestos

Finally, we investigated the association between PCE exposure and the risk of cancer of subsites of the larynx (glottis/subglottis, supraglottis, and other or non-specified). The OR associated with the highest cumulative level of exposure to PCE was higher for cancer of the glottis/subglottis (OR = 5.95; 95%CI [1.73 to 20.53]; five exposed cases) than for cancer of the supraglottis (OR = 3.96; 95%CI [0.72 to 21.78]; two exposed cases) or of other/non-specified subsites (OR = 1.76; 95%CI [0.19 to 16.12]; one exposed case), but the ORs were not significantly different.

## Discussion

In the present study, exposure to chlorinated solvents was not associated with an overall risk of head and neck cancer. However, high exposure to PCE or MC was associated with higher risks of laryngeal and hypopharyngeal cancers, respectively. These results suggest that these commonly used chlorinated solvents may have a deleterious impact on the upper respiratory tract.

Few epidemiological studies have examined the effects of solvents on head and neck cancers, and even fewer the effects of chlorinated solvents on these cancers.

Previous epidemiological studies have most examine exposure to non-specific solvents. In a case-control study, after adjustment for occupational agents and potential non-occupational confounders, a higher risk of both hypopharyngeal and laryngeal cancer was observed for subjects ‘ever exposed’ to solvents relative to those who were not exposed [11]. Another study which considered ‘all chlorinated solvents’ as exposure, also reported an increased risk of laryngeal cancer, although there was no dose-response relationship [13]. To a lesser extent, various other studies have shown a non-significant increased risk of head and neck cancers when solvents were considered overall [8, 10, 21, 22]. However, direct comparison with our results is difficult as the solvent classes were not clearly specified in the previous studies. Indeed, few studies have focused on the specific effect of a single chlorinated solvent on the head and neck.

Among the few studies that have investigated TCE exposure, Blair et al. found a standardized mortality ratio close to the null value for oral cavity and pharyngeal cancers [23]*,* whereas Raaschou-Nielsen et al. found a non-significant increase in the incidence of oral cavity, pharyngeal, and laryngeal cancer among blue-collar workers exposed to TCE [24]. A pooled analysis of three Nordic cohort studies, also showed a non-significant increase in the risk of oral, pharyngeal, and laryngeal cancers [25]. Similar findings were obtained in another study*,* which showed a non-significantly higher hazards ratio for death by cancer of the oral cavity and pharynx among male workers exposed to TCE [26]. Moreover, a non-significant excess of mortality from oral, pharyngeal and laryngeal cancer linked to TCE exposure has also been reported [27]. No information on smoking or alcohol consumption was provided by these studies. In our study, we did not find any association between exposure to TCE and cancer of the oral cavity, pharynx, or larynx after adjustment for smoking, drinking, and exposure to asbestos. An association between exposure to TCE and head and neck cancer risk was reported among women in the ICARE study [17]. In women, confounding by asbestos was minimal and adjustment for asbestos did not modify the estimates related to exposure to TCE and head and neck cancer risk. In contrast, more than 90% of the men exposed to TCE were also exposed to asbestos, and inclusion of asbestos in the models may have led to overadjustment. We repeated the analyses among subjects never exposed to asbestos (479 cases and 1159 controls), and found no association with exposure to TCE (OR = 1.06; 95%CI [0.66 to 1.70]; 55 exposed cases), even if the risk of head and neck cancer increased for those with the highest levels of cumulative exposure (OR = 1.40; 95%CI [0.48 to 4.06]; eight exposed cases). Among men never exposed to asbestos, those exposed to only TCE had a non-significantly elevated risk of head and neck cancer (OR = 1.30; 95%CI [0.74 to 2.27]; 36 exposed cases).

The rare studies that examined PCE exposure in relation with head and neck cancer yielded inconsistent results. Cohort studies reported no increase [28] or a non-significant increase in the frequency of head and neck cancers [29, 30], but were based on a small number of cases, and were not adjusted for tobacco and alcohol consumptions. A small population based case-control study on dry cleaners that examined PCE exposure showed a higher risk of oral cavity or laryngeal cancers in subjects exposed to PCE than those who were not, with some evidence of dose-response and duration-response relationships for laryngeal cancer [14]. Although these results were not significant due to the small number of exposed cases (seven for oral cavity, four for laryngeal cancer), they support our findings, particularly for the association between occupational exposure to PCE and an increased risk of laryngeal cancer. Moreover, the ICARE study has recently shown that exposure to PCE was associated with an increased risk of laryngeal cancer risk in women [17], and an increased risk of lung cancer [16], further supporting a carcinogenic effect of PCE on the respiratory tract. However, there were no studies to suggest mechanisms underlying these effects. Several potential genotoxic and non-genotoxic mechanisms of liver carcinogenesis for perchlorethylene have been identified, but are unlikely to be relevant for respiratory cancer [5]. However, the upper airways are in direct contact with inhaled toxicants, and chronic irritation and inflammation may contribute to the promotion or progression of otherwise initiated lesions. PCE exposure may also facilitate the penetration of other carcinogens in the mucosa, although the lack of statistical interaction in our data between PCE exposure and other risk factors does not support this hypothesis.

Our findings also provide limited evidence of an association between exposure to MC and hypopharyngeal cancer. No other study has specifically examined the risk of head and neck cancer associated with MC exposure [31]. Only two cohort studies of workers exposed to MC reported results for head and neck cancer by subsite, and found either no [32], or a non-significant increased risk for oral cancer [33].

Our study has limitations that may affect the interpretation of the results. First, occupational exposure was retrospectively assessed, and the use of job-exposure matrices generates systematic misclassification, which is likely to be independent of case-control status. Such non differential misclassification could result in an average bias toward the null [34]. Second, the number of exposed cases was small for some chlorinated solvents resulting in large confidence intervals. Investigation of combinations of chlorinated solvents was limited for the same reason and it was not possible to investigate the association between exclusive occupational exposure to PCE and head and neck cancer risk.

Our study also has several strengths. The large number of subjects provided sufficient statistical power to detect moderate associations, and allowed assessment of cancer sites and subsites. Detailed information on lifelong occupational histories was available, allowing us to assess the level of exposure to chlorinated solvents and investigate dose-response relationships. We adjusted for major confounders, such as tobacco and alcohol consumption, as well as exposure to asbestos. Additional adjustments for educational level, socioeconomic status, and exposure to other chlorinated solvents showed that confounding due to these variables was minimal. Selection bias, although always possible, was probably not an important issue in this study. The distribution of the included cases by age and cancer site was similar to that generally observed in France [35]. The lifetime prevalence of exposure to chlorinated solvents among our controls was close to that observed in a representative sample of the French male population [1].

## Conclusions

In summary, our findings suggest that high occupational exposure to PCE may increase the risk of laryngeal cancer. Data from the ICARE study has recently suggested that occupational exposure to PCE also increases the risk of lung cancer [16]. Our results extend these findings by suggesting a likely carcinogenic effect of PCE on other parts of the respiratory tract.

## Additional file

Additional file 1: Figure S1. Image plot for Spearman’s correlations coefficient among chlorinated solvents and asbestos’ CEI. **Table S1.** Associations between exposure to chlorinated solvents and head and neck cancer, without adjustment for asbestos exposure. **Table S2.** Association between head and neck cancer and exposure to chlorinated solvents, with adjustment for educational level. **Table S3.** Association between head and neck cancer and exposure to chlorinated solvents, with adjustment for occupational class. (PDF 263 KB)

## Abbreviations

CEI: Cumulative exposure index
CF: Chloroform
CI: Confidence interval
CT: Carbon tetrachloride
MC: Methylene chloride
OR: Odds-ratio
PCE: Perchloroethylene
TCE: Trichloroethylene
