# Supplementary material for: Occupational exposure to chlorinated solvents and risk of head and neck cancer in men: a population-based case-control study in France
Source: Environ Health. 2017 Jul 24;16:77. doi: 10.1186/s12940-017-0286-5 (PMC5525363; doi:10.1186/s12940-017-0286-5)
Supplement: Additional file 1: Figure S1. — Image plot for Spearman’s correlations coefficient among chlorinated solvents and asbestos’ CEI. Table S1. Associations between exposure to chlorinated solvents and head and neck cancer, without adjustment for asbestos exposure. Table S2. Association between head and neck cancer and exposure to chlorinated solvents, with adjustment for educational level. Table S3. Association between head and neck cancer and exposure to chlorinated solvents, with adjustment for occupational class. (PDF 263 KB) [file 12940_2017_286_MOESM1_ESM.pdf]

Figure S1. Image plot for Spearman's correlations coefficient among chlorinated solvents and asbestos' CEI

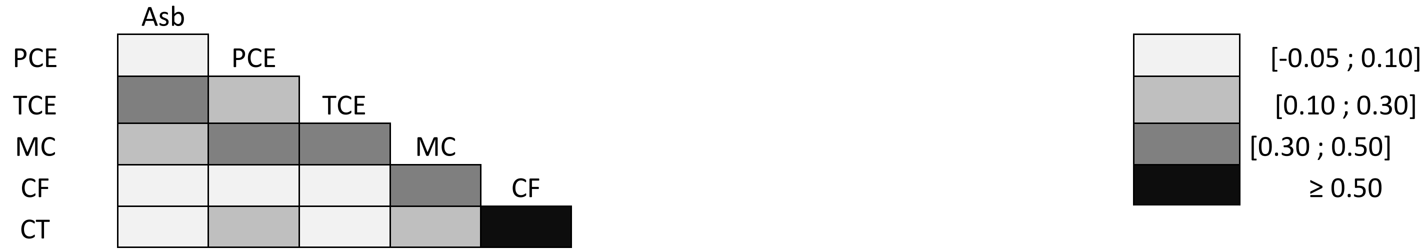

Abbreviations : Asb, asbestos; PCE, perchloroethylene; TCE, trichloroethylene; MC, methylene chloride ; CF, chloroform; TC, carbon tetrachloride

Table S1. Associations between exposure to chlorinated solvents and head and neck cancer, without adjustment for asbestos exposure

	PCE			TCE			MC			CF			TC			At least one of these five chlorinated solvents		
**Exposure**																		
	**Co**	**Ca**	**OR[1] [95%CI]**	**Co**	**Ca**	**OR[1] [95%CI]**	**Co**	**Ca**	**OR[1] [95%CI]**	**Co**	**Ca**	**OR[1] [95%CI]**	**Co**	**Ca**	**OR[1] [95%CI]**	**Co**	**Ca**	**OR[1] [95%CI]**
Never	2670	1747	1	1745	998	1	2518	1618	1	2712	1808	1	2715	1802	1	1704	990	1
Ever	94	76	1.14 [0.75 ; 1.73]	1024	830	1.20 [1.02 ; 1.42]	246	206	1.04 [0.80 ; 1.35]	52	15	0.58 [0.27 ; 1.23]	49	21	0.61 [0.30 ; 1.21]	1065	838	1.18 [1.02 ; 1.38]
**Duration[2]**																		
Short	44	33	1.12 [0.63 ; 2.16]	301	227	1.10 [0.85 ; 1.42]	99	90	1.12 [0.76 ; 1.65]	22	7	0.77 [0.27 ; 2.20]	26	10	0.59 [0.23 ; 1.50]	303	225	1.10 [0.85 ; 1.42]
Intermediate	26	25	1.40 [0.65 ; 3.01]	401	312	1.26 [1.01 ; 1.58]	67	50	0.85 [0.51 ; 1.40]	13	3	0.21 [0.03 ; 1.38]	10	4	0.35 [0.06 ; 1.99]	420	315	1.21 [0.97 ; 1.51]
Long	24	18	0.87 [0.38 ; 1.96]	322	290	1.24 [0.98 ; 1.57]	80	66	1.11 [0.71 ; 1.73]	17	5	0.63 [0.16 ; 2.38]	13	7	0.85 [0.25 ; 2.91]	342	297	1.24 [0.95 ; 1.53]
p for trend			0.93			0.05			0.77			0.19			0.38			0.08
**CEI**																		
Low	47	39	1.29 [0.72 ; 2.30]	515	414	1.22]1.00 . 1.49]	121	109	1.18 [0.82 ; 1.69]	26	5	0.51 [0.15 ; 1.71]	25	7	0.45 [0.16 ; 1.33]	536	419	1.22 [0.99 ; 1.49]
Medium	36	21	0.71 [0.34 ; 1.51]	409	339	1.25 [1.00 ; 1.56]	101	68	0.72 [0.47 ; 1.10]	26	10	0.62 [0.23 ; 1.66]	24	14	0.75 [0.30 ; 1.85]	423	318	1.12 [0.90 ; 1.40]
High	11	16	1.79 [0.68 ; 4.72]	100	76	1.01 [0.68 ; 1.50]	24	29	1.75 [0.87 ; 3.55]							106	100	1.19 [0.82 ; 1.73]
p for trend			0.42			0.50			0.47			0.14			0.41			0.30

Abbreviations: PCE=perchloroethylene; TCE=trichloroethylene; MC=methylene chloride; CF=chloroform; CT=carbon tetrachloride; OR= odds ratio; CI= confidence interval; CEI= cumulative exposure index; Ca= cases; Co= controls

1-OR adjusted for age at interview, residence area, alcohol consumption, smoking status, frequency and duration of smoking

2- Categories of duration of exposure to solvents were defined as follows: for PCE: short : < 5, intermediate: 5-15, long : > 15; for TCE and 'at least to one chlorinated solvent': short: <5, intermediate : 5-20, long : > 20; for MC and CT short : < 5, intermediate : 5 to 15, long : > 15; for CF short : < 10, intermediate : 10 to 20, long : > 20

Table S2. Association between head and neck cancer and exposure to chlorinated solvents, with adjustment for educational level

	PCE			TCE			MC			CF			CT			At least one chlorinated solvent		
**Exposure**	**Co**	**Ca**	**OR[1] [95%CI]**	**Co**	**Ca**	**OR[1] [95%CI]**	**Co**	**Ca**	**OR[1] [CI95%]**	**Co**	**Ca**	**OR[1] [CI95%]**	**Co**	**Ca**	**OR[1] [CI95%]**	**Co**	**Ca**	**OR [95%CI]**
Never	2498	1445	1	1646	808	1	2354	1323	1	2534	1494	1	2537	1490	1	1605	801	1
Ever	86	63	0.93 [0.60 to 1.44]	943	703	0.97 [0.80 to 1.19]	230	185	0.92 [0.70 to 1.21]	50	14	0.83 [0.38 to1.83]	47	18	0.71 [0.35 to 1.47]	984	710	0.96 [0.79 to1.17]
**Duration[2]**																		
Short	40	29	1.03 [0.54 to 1.94]	275	194	0.86 [0.66 to 1.14]	89	83	1.05 [0.70 to 1.56]	20	2	0.94 [0.31 to 2.82]	24	10	0.71 [0.27 to 1.90]	277	193	0.95 [0.71 to 1.26]
Intermediate	23	18	0.94 [0.41 to 2.16]	373	271	0.97 [0.76 to 1.24]	63	40	0.65 [0.38 to 1.11]	13	1	0.22 [0.03 to 1.63]	10	3	0.52 [0.10 to 2.83]	392	274	0.98 [0.76 to 1.27]
Long	23	16	0.78 [0.35 to 1.76]	295	238	0.95 [0.73 to 1.23]	78	62	0.99 [0.62 to 1.57]	17	2	0.91 [0.24 to 3.49]	13	5	0.88 [0.23 to 3.29	315	243	0.93 [0.71 to 1.22]
**CEI**																		
Low	43	32	1.06 [0.58 to 1.95]	473	357	1.01 [0.80 to 1.28]	109	97	1.05 [0.71 to 1.54]	25	3	0.93 [0.28 to 3.08]	23	7	0.53 [0.17 to 1.62]	495	363	1.02 [0.81 to 1.29]
Medium	32	19	0.58 [0.27 to 1.24]	378	285	0.98 [0.79 to 1.26]	97	61	0.64 [0.41 to 0.99]	25	2	0.77 [0.27 to 2.15]	24	11	0.89 [0.35 to 2.26	388	263	0.89 [0.69 to 1.15]
High	11	12	1.49 [0.54 to 4.11]	92	61	0.79 [0.52 to 1.21]	24	27	1.5 [0.73 to 3.06]							101	84	0.93 [0.63 to 1.38]

Abbreviations: PCE=perchloroethylene; TCE=trichloroethylene; MC=methylene chloride; CF=chloroform; CT=carbon tetrachloride; OR= odds ratio; CI= confidence interval; CEI= cumulative exposure index; Ca= cases; Co= controls

1-OR adjusted for age at interview, residence area, alcohol consumption, smoking status, frequency and duration of smoking, exposure to asbestos and level of education

2- Categories of duration of exposure to solvents were defined as follows: for PCE: short : < 5, intermediate: 5-15, long : > 15; for TCE and 'at least to one chlorinated solvent': short: <5, intermediate : 5-20, long : > 20; for MC and CT short : < 5, intermediate : 5 to 15, long : > 15; for CF short : < 10, intermediate : 10 to 20, long : > 20

Table S3. Association between head and neck cancer and exposure to chlorinated solvents, with adjustment for occupational class

	PCE			TCE			MC			CF			CT			At least one chlorinated solvent		
**Exposure**	**Co**	**Ca**	**OR[1] [95%CI]**	**Co**	**Ca**	**OR[1] [95%CI]**	**Co**	**Ca**	**OR[1] [95%CI]**	**Co**	**Ca**	**OR[1] [95%CI]**	**Co**	**Ca**	**OR[1] [95%CI]**	**Co**	**Ca**	**OR[1] [95%CI]**
Never	2581	1635	1	1686	938	1	2432	1508	1	2619	1691	1	2622	1686	1	1645	930	
Ever	89	70	0.95 [0.62 to 1.45]	989	770	0.84 [0.69 to 1.02]	238	197	0.87 [0.66 to 1.13]	51	14	0.73 [0.33 to 1.63]	48	19	0.69 [0.34 to 1.41]	1030	778	0.84 [0.69 to 1.01]
**Duration[2]**																		
Short	41	31	1 [0.53 to 1.86]	289	215	0.84 [0.63 to 1.11]	93	88	0.92 [0.62 to 1.36]	21	7	0.94 [0.31 to 2.82]	25	10	0.63 [0.24 to 1.67]	291	213	0.86 [0.65 to 1.13]
Intermediate	25	23	1.13 [0.53 to 2.44]	390	290	0.89 [0.70 to 1.15]	66	46	0.71 [0.43 to 1.19]	13	2	0.22 [0.03 to 1.63]	10	3	0.43 [0.07 to 2.50]	409	295	0.87 [0.68 to 1.12]
Long	23	16	0.79 [0.32 to 1.61]	310	265	0.78 [0.60 to 1.02]	79	63	0.92 [0.59 to 1.44]	17	5	0.91 [0.24to 3.49]	13	6	1.05 [0.30 to 3.63]	330	270	0.78 [0.60 to 1.01]
**CEI**																		
Low	44	36	1.1 [0.61 to 1.99]	497	391	0.90 [0.71 to 1.13]	114	107	1 [0.69 to 1.45]	25	5	0.8 [0.23 to 2.78]	24	7	0.51 [0.16 to 1.55]	518	397	0.93 [0.74 to 1.17]
Medium	34	20	0.58 [0.27 to 1.24]	397	313	0.83 [0.64 to 1.06]	100	63	0.62 [0.40 to 0.95]	26	9	0.69 [0.25 to 1.94]	24	12	0.87 [0.34 to 2.18]	408	291	0.75 [0.58 to 0.96]
High	11	14	1.39 [0.53to 3.65]	95	66	0.64 [0.42 to 10.97]	24	27	1.27 [0.62 to 2.58]							104	90	0.76 [0.51 to 1.11]

Abbreviations: PCE=perchloroethylene; TCE=trichloroethylene; MC=methylene chloride; CF=chloroform; CT=carbon tetrachloride; OR= odds ratio; CI= confidence interval; CEI= cumulative exposure index; Ca= cases; Co= controls.

1-OR adjusted for age at interview, residence area, alcohol consumption, smoking status, frequency and duration of smoking, exposure to asbestos and category of the longest job held

2- Categories of duration of exposure to solvents were defined as follows: for PCE: short : < 5, intermediate: 5-15, long : > 15; for TCE and 'at least to one chlorinated solvent': short: <5, intermediate : 5-20, long : > 20; for MC and CT short : < 5, intermediate : 5 to 15, long : > 15; for CF short : < 10, intermediate : 10 to 20, long : > 20.
